# Geographic Variability, Seasonality, and Increase in ASPCA Animal Poison Control Center Harmful Blue-Green Algae Calls—United States and Canada, 2010–2022

**DOI:** 10.3390/toxins15080505

**Published:** 2023-08-15

**Authors:** Rebecca A. Bloch, Grace Faulkner, Elizabeth D. Hilborn, Tina Wismer, Nicole Martin, Sarah Rhea

**Affiliations:** 1 College of Veterinary Medicine, Department of Population Health and Pathobiology, North Carolina State University, Raleigh, NC 27606, USA; rabloch@ncsu.edu (R.A.B.); gfaulkn2@ncsu.edu (G.F.); hilborn.e@epa.gov (E.D.H.); 2 Center for Public Health and Environmental Assessment, Office of Research and Development, United States Environmental Protection Agency, Chapel Hill, NC 27514, USA; 3 American Society for the Prevention of Cruelty to Animals, Animal Poison Control Center, Champaign, IL 61820, USA; tina.wismer@aspca.org (T.W.); nicole.martin@aspca.org (N.M.)

**Keywords:** harmful blue-green algae, ASPCA Animal Poison Control Center, surveillance, cyanobacteria, sentinels

## Abstract

Harmful cyanobacteria (blue-green algae) exposures can cause illness or death in humans and animals. We characterized American Society for the Prevention of Cruelty to Animals (ASPCA) Animal Poison Control Center (APCC) harmful blue-green algae (HBGA) call data, compared it to a measure of harmful algal bloom public awareness, and considered its suitability as a public health information source. ASPCA APCC dog and cat “HBGA exposure” calls made 1 January 2010–31 December 2022 were included. We calculated annual HBGA call percentages and described calls (species, month, origin, exposure route). We characterized public awareness by quantifying Nexis Uni^®^ (LexisNexis Academic; New York, NY, USA)-indexed news publications (2010–2022) pertaining to “harmful algal bloom(s)”. Call percentage increased annually, from 0.005% (2010) to 0.070% (2022). Of 999 HBGA calls, 99.4% (*n* = 993) were dog exposures. Over 65% (*n* = 655) of calls were made July–September, largely from the New England (*n* = 154 (15.4%)) and Pacific (*n* = 129 (12.9.%)) geographic divisions. Oral and dermal exposures predominated (*n* = 956 (95.7%)). Harmful algal bloom news publications increased overall, peaking in 2019 (*n* = 1834). Higher call volumes in summer and in the New England and Pacific geographic divisions drove HBGA call increases; public awareness might have contributed. Dogs and humans have similar exposure routes. ASPCA APCC HBGA call data could serve as a public health information source.

## 1. Introduction

Harmful algal blooms, including those caused by cyanobacteria or blue-green algae, are a persistent problem in the United States (US) [1,2] and worldwide [2,3], showing increases in some locations [4]. This is due, in part, to rising global temperatures, elevated nutrient contamination and carbon dioxide levels in aquatic environments, and a surge in freshwater usage [2]. Harmful blue-green algae (HBGA) are typically responsible for freshwater harmful algal blooms and can also be found in marine environments [5]. Exposure to HBGA can cause illness or death in invertebrates [6], fish [7,8], birds [1], and mammals, including humans [9], companion animals [10], livestock [11], and wildlife [1,12]. Human HBGA exposures occur through the ingestion of contaminated shellfish [13], the ingestion of or dermal contact with contaminated water [9], or respiratory exposure to aerosolized harmful algal bloom toxins [14]. Dog HBGA exposures occur in similar ways, but with grooming behavior as an added route of cyanobacterial ingestion [10]. Compared to humans, dogs are less discerning of the water they swim in and drink and have been observed to consume dried cyanobacterial mats [15]. Although dependent on the toxin amount and type and route of exposure, clinical signs of HBGA exposure in dogs, including gastrointestinal distress and neurological signs, can develop within hours, and exposure can result in death [10].

Surveillance efforts to prevent HBGA exposures have been undertaken mainly on state and local levels [16], although federally led efforts towards broader surveillance exist. The National Centers for Coastal Ocean Science (NCCOS) monitors US coastal and lake regions for harmful algal blooms with near-real-time bloom detection [17]. The Centers for Disease Control and Prevention’s (CDC’s) One Health Harmful Algal Bloom System (OHHABS) [18] is the only comprehensive human and animal health-related harmful algal bloom surveillance system in the country but is limited to voluntary reporting by states and US territories and is not representative of the entire US.

Sentinels, or indicators of harmful algal bloom presence, are another way to monitor HBGA. Companion animals (e.g., dogs, cats) and other animal species have acted as sentinels for numerous environmental exposures and cancer outcomes [19]. On multiple occasions, HBGA-associated illness or death in animals, including companion animals, preceded human illness and served as sentinel events and warnings of potential health risk [20]. Studies characterizing companion animal HBGA exposures have a variety of data sources. These have included published reports [10,20]; data from a legacy (i.e., closed) HBGA human and animal health surveillance system [10,21]; veterinary medical record data from a single, large veterinary teaching hospital [10]; and HBGA-related dog illnesses and deaths reported to a single state health department [9].

The American Society for the Prevention of Cruelty to Animals (ASPCA) maintains the Animal Poison Control Center (APCC) emergency fee-based poison control hotline which assists the public, veterinarians, and other animal care professionals across the US and Canada to identify and treat intoxications in animals [22]. From each call, the ASPCA APCC collects data, coded by toxicant and maintained in their proprietary database system, AnTox, for toxicant diagnosis and treatment [22]. Call data have been used to describe companion animal exposures to potentially toxic substances, including call trends over time by high-level toxicant categories [23,24] and focused studies of calls related to specific human and veterinary prescription and non-prescription medications, insecticides, foods, and other household products [23,25,26]. ASPCA APCC HBGA calls have never been described in the published literature. The objectives of this study were to (1) characterize 13 years of ASPCA APCC HBGA calls (2010–2022), including identification of changes in HBGA call frequency, over time and by season and geographic division (or region), and in annual percentages of HBGA calls compared to all ASPCA APCC calls; (2) evaluate these data as a reflection of public health awareness of harmful algal blooms; and (3) consider the suitability of these data as a public health information source.

## 2. Results

During 1 January 2010–31 December 2022, a total of 999 HBGA calls meeting our inclusion criteria were made in the US or Canada to ASPCA APCC (Table 1). By year, the mean and median number of HBGA calls from all sources were 77 and 41, respectively, with a range of nine (2010) to two-hundred-and-ten (2021). The mean and median total annual calls to the ASPCA APCC were 211,219 and 181,818, respectively, and ranged from 165,900 to 320,350 per year.

**Figure 1:**
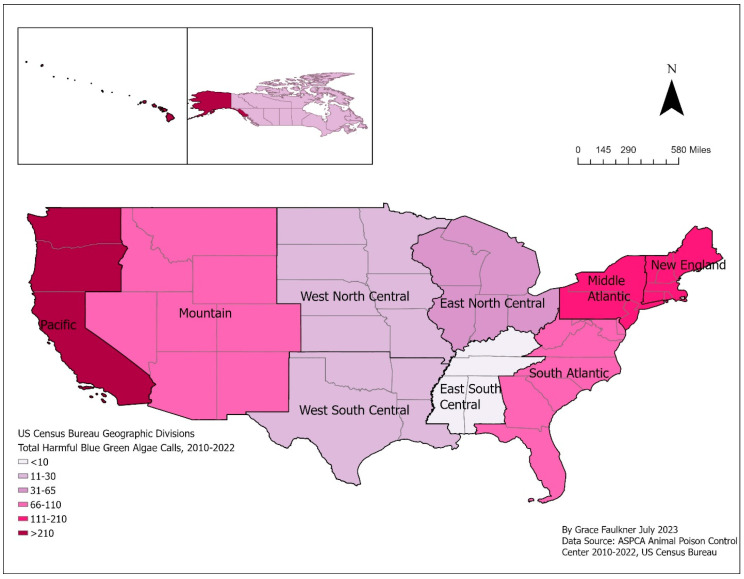
Distribution and frequency of harmful blue-green algae exposure calls made to American Society for Prevention of Cruelty to Animals (ASPCA) Animal Poison Control Center (APCC) in the US and Canada, 2010–2022. Calls were grouped by US geographic division, as defined by the US Census Bureau [27], and Canada. The map shows the continental US while the inset map shows Hawaii and Alaska, both considered part of the Pacific region, and Canada. The color of the region indicates the total number of calls received in that region. Inset maps are not to scale.

Almost 98% (979) of HBGA calls came from the US, and 2% (*n* = 20) came from Canada (Table 1 and Figure 1). Calls from Canada were located close to the US border in Ontario, Quebec, and British Columbia. 

**Figure 2:**
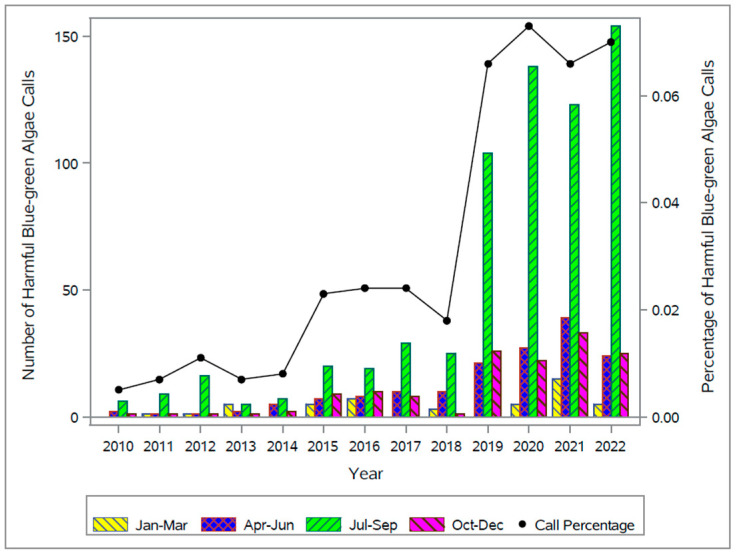
Percentage of harmful blue-green algae exposure calls out of all calls made to the American Society for the Prevention of Cruelty to Animals (ASPCA) Animal Poison Control Center (APCC) Per Year and the number of harmful blue-green algae exposure calls by quarter in the United States and Canada, 2010–2022.

The annual percentage of HBGA calls (the percentage of HBGA calls per year out of the total number of calls to the ASPCA APCC for that year) increased from 0.005% (2010) to 0.070% (2022), peaking at 0.073% (2020) (Figure 2). The largest increase in annual HBGA call percentage from one year to the following year occurred from 0.018% (*n* = 39) in 2018 to 0.066% (*n* = 151) in 2019. The second largest increase in annual HBGA call percentage occurred from 0.008% (*n* = 14) in 2014 to 0.023% (*n* = 41) in 2015; the majority of these occurred July–September.

**Figure 3:**
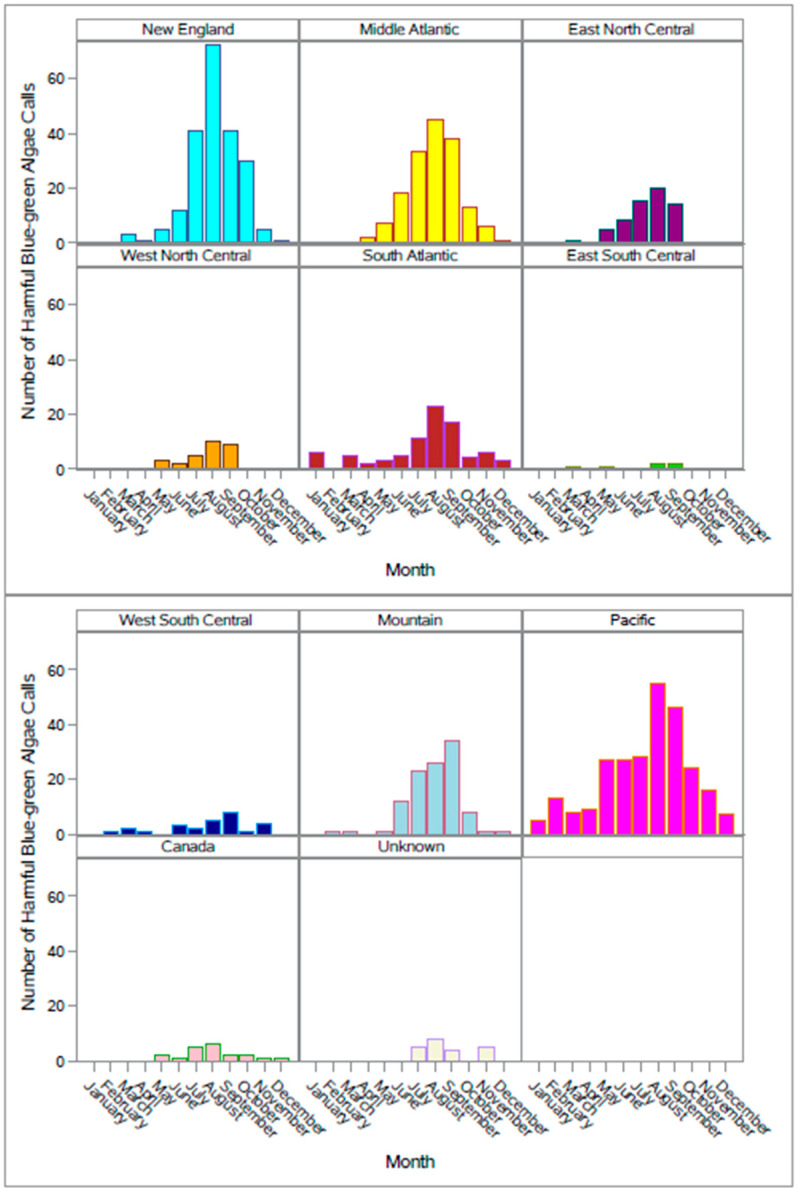
Number of harmful blue-green algae exposure calls per month made to the American Society for the Prevention of Cruelty to Animals (ASPCA) Animal Poison Control Center (APCC) by geographic divisions of the United States, 2010–2022. Each month represents the total number of harmful blue-green algae exposure calls for that month for all years. Call geographic divisions were based on the US Census Bureau nine geographic divisions [27]; calls from Canada were grouped Canada; calls without a recorded call location were grouped “Unknown”.

Across the 13-year period, 65.6% of calls were made July–September (655 HBGA calls July–September/999 HBGA calls all months). Between the months of July and September during the study period (2010–2022), 40% (399/999) of all HBGA calls were from the New England (*n* = 154 (15.4%)), Pacific (*n* = 129 (12.9%)), and Mid-Atlantic (*n* = 116 (11.6%)) geographic divisions, peaking in August (Figure 3). Further variability occurred among different geographic divisions. For example, calls peaked in September in the Mountain and West South Central divisions, while calls in all other geographic divisions peaked in August (Figure 2). In addition, over half of the HBGA calls from the New England (158/211) and Pacific (150/265) divisions occurred during 2020–2022. 

Ninety-nine percent (*n* = 993) of HBGA calls were dog exposures, while six calls were cat exposures with five of these coming from the same call location on the same day (Table 1). Over 95% (956/999) of HBGA call exposures were either dermal and oral, or oral only. Regarding agent certainty, just over 60% (603/999) of HBGA calls were “observed” or “evidence”, and 8.5% (85/999) were “suspected”.

**Figure 4:**
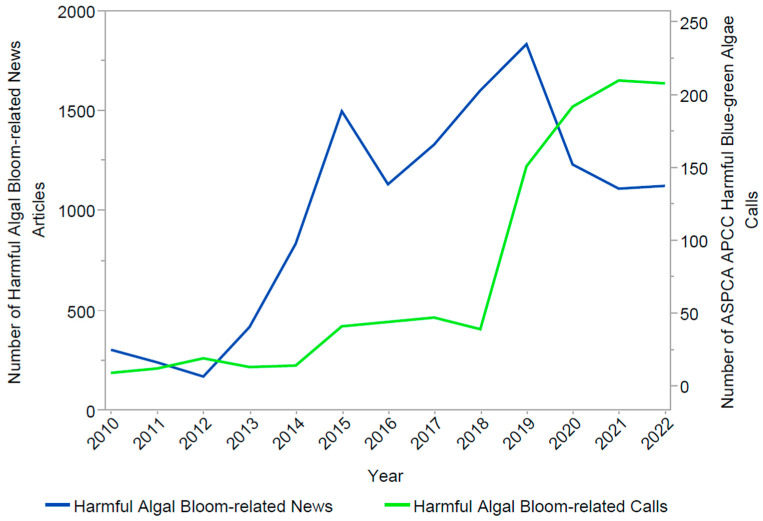
Annual Nexis Uni^®^-indexed news publications containing the term “harmful algal bloom” published in the US, 2010–2022, and number of harmful blue-green algae exposure calls made to the American Society for Prevention of Cruelty to Animals (ASPCA) Animal Poison Control Center (APCC) in the US and Canada, 2010–2022. Nexis Uni^®^ results represent numbers of news articles published annually.

Nexis Uni^®^-indexed news publications containing the term “harmful algal bloom” increased from 2010 (*n* = 301) to 2022 (*n* = 1123). News publication peaks occurred in 2015 (*n* = 1499) and 2019 (*n* = 1834) (Figure 4).

## 3. Discussion

Historically, a variety of animal species have served as environmental sentinels to warn of human health risks [28]. With their close contact to and shared recreational risks with people, companion animals, particularly dogs, might be uniquely suited as environmental sentinels [19,20,29]. While harmful algal blooms are often characterized by turbid, colored water, or by algal mats or surface scums, toxins can be present without visual evidence of a potential health risk [20,30]. When harmful algal blooms are not recognized, dog illness might be an early indicator of potential human health risk [20].

In this study, companion animal HBGA calls to ASPCA APCC increased dramatically during 2010–2022, starting with a smaller increase from 2014 to 2015 and then a much larger increase from 2018 to 2019. Annual percentage increases in ASPCA APCC HBGA calls were almost exclusively due to the July–September call volume growth, a mid-to-late summer seasonal pattern observed beginning in 2018. A similar pattern is observed in OHHABS reported human illnesses which peaked in July (2019) and August (2020) [31,32].

The 2010–2022 ASPCA APCC HBGA call increase is likely the result of multiple factors. As the climate changes [33,34], harmful algal blooms are increasingly reported in the US [35] and globally [3,36]. Therefore, it is plausible that HBGA exposures among companion animals did indeed increase during the study period. The observed increase in HBGA calls could reflect a shift over time in the distribution of caller type, from calls made by veterinarians to an increasing majority of calls made directly to ASPCA APCC by companion animal owners. Although caller type was not available for our analysis, in a study of all ASPCA APCC intoxications data from 2005–2014, Shahin, et al. reported that 70% of calls were made by companion animal owners [37], not veterinarians. This finding suggests an expanding role for animal poison control tele-triage services [25] and possible changes in public awareness of animal toxicant exposures overall. The general increase in harmful algal bloom news publications during 2010–2022 also suggests that public awareness of harmful algal blooms and their potential associated health effects have been on the rise. The 2015 and 2019 peaks in the “harmful algal bloom” Nexis Uni^®^ publications (Figure 4) correspond to the largest increases in ASPCA APCC HBGA calls. National reporting of events like the Lake Erie HBGA in 2014 that “left nearly 400,000 people in Ohio without drinking water for 2 days” [38] and three dog deaths in North Carolina [39], followed by eight additional dog deaths in Michigan from harmful algal blooms in August 2019 [40], could have contributed to public awareness. 

Most of the 2010–2022 ASPCA APCC HBGA calls made in the US originated from the New England and Pacific divisions, with fewer of these calls originating from the Mid- and South Atlantic divisions (Table 1). The New England and Pacific divisions are both coastal locations with fresh and saltwater environments. Harmful algal blooms are most often associated with freshwater, though their presence in salt or brackish water is also possible [5]. At least 75% of harmful algal blooms reported to OHHABS are in freshwater sources, primarily reservoirs and lakes [31,32]. Freshwater areas, like Lake Erie, and brackish water areas, like Lake Pontchartrain, are monitored closely by NCCOS for harmful algal blooms [17]. Information on water sources associated with the HBGA calls was not available for this study.

Veterinarians serve an important public health role by recognizing possible HBGA-associated illness in animals that could forewarn of HBGA bloom development detrimental to people or other animals. Veterinarians’ awareness of and ability to help mitigate this emerging public health issue could be enhanced through additional education on the signs associated with HBGA exposure in animals and connection to local or state officials who can receive reports of suspected HBGA exposures. In the months following increased public health outreach to medical personnel and veterinarians in the state of Kansas, 13 human and animal exposures were identified, where there had been none the previous year [9]. 

Dog HBGA-associated illness can be a sentinel and a warning for human health risk. Dogs frequently accompany people to locations where HBGA might be encountered, and their exposure routes are similar to those of humans [41]. For example, the primary exposure routes among human harmful algal bloom toxicities self-reported to poison control centers were dermal and oral, similar to companion animal HBGA calls to ASPCA APCC (Table 1) [42]. Although information on clinical signs was not available in the ASPCA APCC HBGA call data analyzed here, dogs exposed to harmful algal blooms commonly exhibit gastrointestinal signs [21], matching this commonly affected system in humans who are exposed to harmful algal blooms [42]. While dogs are appropriate sentinels of HBGA, consideration for the use of these data would require agreement between state or local officials and the ASPCA APCC, as well as routine collection in a timely manner to be of use in the protection of human and animal health from further harm.

Changing climatic conditions will likely continue to contribute to an increase in harmful algal blooms caused by an assortment of opportunistic organisms, including HBGA, which thrive in the warm, stagnant water [2] frequently present in mid-to-late summer in many US locations. This timing coincides with summer recreational activities and behaviors that put people and their animals in close contact with potentially contaminated waters. Approximately 60% of reported harmful algal bloom animal events in CDC’s OHHABS occur in parks or public recreational areas [32], suggesting a possible shared risk with pet owners and other park visitors. At a national level, OHHABS has integrated human and animal harmful algal bloom reports across several states and jurisdictions. However, the true harmful algal bloom exposure rate is unknown and could be higher with the contribution of data to OHHABS from additional states. Increased participation in harmful algal bloom surveillance across human, animal, and environmental sectors could help to maximize the response to harmful algal blooms across the US. 

This study has several limitations. Companion animal HBGA exposures and illnesses that did not result in an ASPCA APCC call were not included; available data are restricted to situations in which ASPCA APCC services and consultation were sought and paid for. Previous examination of the ASPCA APCC data found an association between high call areas and areas of high socioeconomic status [43]. As a fee-based service, consultation may not be undertaken equally by all people associated with animals experiencing HBGA exposures. Other reasons that companion animal HBGA exposures would not be captured in these ASPCA APCC data include situations in which an animal died acutely or if an animal did not exhibit clinical signs, or clinical signs were not severe enough that expert toxicologic assistance was required. Of all human illnesses related to harmful algal bloom events in 2020 that were captured by OHHABS, 77% resulted in the person seeking health care; among those who sought healthcare, only 71% called a human poison control center for guidance [31]. Despite these limitations, almost 69% of HBGA calls in this analysis were “observed”, “evidence”, or “suspected” with regard to certainty that the agent of exposure was HBGA. Just over 2% of HBGA calls originated from Canada, which does not have an animal poison control center. It was not possible to exclude these calls from analysis because the total annual number of calls for HBGA or any toxicant were not available by country of origin. Toxin concentration, specific toxin testing, or follow-up treatments and outcomes associated with HBGA calls were not available for this analysis. HBGA itself can be associated with adverse health effects in the absence of cyanobacterial toxin concentrations known to be associated with illness. Because HBGA occur in aquatic assemblages of organisms, it is possible that pathogens, fungal toxins, irritants, or other uncharacterized bioactive compounds present in the aquatic community may be associated with the nonspecific adverse health effects reported among animals after exposure to HBGA [44]. 

## 4. Conclusions

The frequency and annual percentage of companion animal HBGA exposure calls from the US and Canada and in the AnTox database increased during 2010–2022. Dogs may accompany humans to HBGA-contaminated locations and, like humans, are typically exposed via oral and dermal routes. Education and public awareness through educational campaigns and media are important components for increasing harmful algal bloom awareness and reporting. The integration of ASPCA APCC HBGA calls of dog HBGA-associated illnesses and deaths may be a source of locational information for local and state officials. If routinely collected and used in a timely manner, animal HBGA-associated illnesses and deaths could provide early warning of potential harmful algal bloom events and inform human and animal harmful algal bloom exposure prevention efforts. The inclusion of animal poison control HBGA calls may also be used to inform outbreak investigation of suspected HBGA-associated illness [45].

## 5. Materials and Methods

Information was compiled from the ASPCA APCC computer database (AnTox, ASPCA, New York City, NY, USA) of phone calls made from throughout the US and Canada. As a 24 h veterinary toxicology service, the ASPCA APCC collects information on animal species, breed, age, sex, weight; number of animals at risk and number of animals affected; time of ingestion; and onset, severity, and duration of clinical signs. Trained call respondents determine the level of suspicion of exposure and the certainty of the toxicant or agent. 

Suspicion of exposure: Each call (or case) was assessed as a “high”, “medium”, or “low” suspicion of exposure to HBGA or “doubtful exposure” to HBGA. “High” suspicion of exposure was recorded when the animal’s clinical signs were consistent with the expected effects of HBGA. “Medium” suspicion of exposure was recorded when the animal’s clinical signs were consistent with the expected effects of HBGA but some exposure data were lacking. “Low” suspicion of exposure was recorded when the animal’s clinical signs or the reported time of ingestion were incompatible with HBGA ingestion. “Doubtful exposure” was recorded when the animal’s clinical history and clinical signs were not consistent with the effects of HBGA. 

Agent certainty: Each call was also assessed for “agent certainty”, or the certainty about the identity of the agent in question which, for this analysis, was HBGA. This designation is based on a rubric that defines the agent as “observed”, “evidence”, “suspected”, “possible”, “not specific”, or “unknown” (Box 1).

Box 1 **Definitions of agent certainty, American Society for the Prevention of Cruelty to Animals, Animal Poison Control Center.**

Observed: Caller has original packaging, label, or image from receipt

Evidence: Caller has majority of information on package, label, receipt, image, but some identifiers are missing

Suspected: Caller believes this is the agent involved based on partial identifiers or how the animal is acting

Possible: Caller knows the product but not the specific ingredients, or observed a potential exposure (e.g., licking a puddle of unknown liquid by a car) such that all potential agents the animal could have had access to (e.g., antifreeze, brake fluid) receive this designation

Not specific: Agent is known but concentration is not known, or the agent indicated in the call record has the same ingredients but is not the exact product

Unknown: Information to designate agent certainty is unknown

Final HBGA calls dataset for analysis: From all calls made during 1 January 2010–31 December 2022 in the US or Canada to the ASPCA APCC and in the AnTox database, we selected calls with a “high” or “medium” suspicion of HBGA exposure. Of these “high” or “medium” suspicion of HBGA exposure calls, we subsequently excluded calls that referred to blue-green algae health supplement exposure, as supplement exposure was not the focus of this analysis. The remaining “high” or “medium” HBGA exposure calls were included in the final dataset for analysis.

Although alternatives to the term “HBGA” were considered for this report, the term blue-green algae was retained for consistency with the way it is encoded in the ASPCA APCC database. The designation of “harmful” was needed to differentiate from ambient blue-green algae or cyanobacteria accumulations that were not reported to be associated with adverse health effects.

Identity of the caller (e.g., companion animal owner, veterinarian), time of ingestion, and information on clinical course were unavailable for this analysis. Individual callers could report one or more animal exposures from the same location on the same day (e.g., two dogs from the same household with the same exposure). In this situation, each animal exposure was a separate case and, therefore, considered a unique call.

We calculated annual percentages of HBGA calls using yearly totals of all calls (i.e., calls related to any potential toxicant in any species) made in the US or Canada to ASPCA APCC and in the AnTox database. Descriptive information about the HBGA calls, including animal species, exposure route, agent certainty, and, for US calls, geographic division (or region), was summarized by frequency and percent. Call geographic divisions were based on the US Census Bureau’s nine geographic divisions [27]; calls from Canada were grouped “Canada”.

We assessed public interest in and awareness of HBGA by searching Nexis Uni^®^ (LexisNexis Academic; New York City, USA) for the term “harmful algal bloom” in news reporting published in the US during 2010–2022. Nexis Uni^®^ results represent numbers of news articles published annually for the included years. 

Analyses were performed using SAS (SAS Version 9.4, 2016; SAS Institute Inc., Cary, NC, USA), JMP (JMP Pro Version 16.0.0, 2021; SAS Institute Inc., Cary, NC, USA), and Excel (Microsoft Office Version 16, 2023; Redmond, WA, USA).

## Figures and Tables

**Table 1:** Characteristics of harmful blue-green algae exposure calls made in the United States and Canada to American Society for the Prevention of Cruelty to Animals (ASPCA) Animal Poison Control Center (APCC) by month of call, 2010–2022.

	January–March (*n* = 47)	April–June (*n* = 157)	July–September (*n* = 655)	October–December (*n* = 140)	Total ^b^ (*n* = 999)
Geographic Division ^a^ *n* (%)					
New England	3 (6.4)	18 (11.5)	154 (23.5)	36 (25.7)	211 (21.1)
Mid Atlantic	0	27 (17.2)	116 (17.7)	20 (14.3)	163 (16.3)
East North Central	1 (2.1)	13 (8.3)	49 (7.5)	0	63 (6.3)
West North Central	0	5 (3.2)	24 (3.7)	0	29 (2.9)
South Atlantic	11 (23.4)	10 (6.4)	51 (7.8)	13 (9.3)	85 (8.5)
East South Central	1 (2.1)	1 (0.6)	4 (0.6)	0	6 (0.6)
West South Central	3 (6.4)	4 (2.6)	15 (2.3)	5 (3.6)	27 (2.7)
Mountain	2 (4.3)	13 (8.3)	83 (12.7)	10 (7.1)	108 (10.8)
Pacific	26 (55.3)	63 (40.1)	129 (19.7)	47 (33.6)	265 (26.5)
Canada	0	3 (1.9)	13 (2.0)	4 (2.9)	20 (2.0)
Unknown	0	0	17 (2.6)	5 (3.6)	22 (2.2)
Exposure route ^c^ *n* (%)					
Dermal and oral	17 (36.2)	61 (38.9)	335 (51.2)	57 (40.7)	470 (47.0)
Oral	30 (63.8)	86 (54.8)	293 (44.7)	77 (55.0)	486 (48.6)
Dermal	0	4 (2.6)	14 (2.1)	3 (2.1)	21 (2.1)
Other	0	0	2 (0.3)	0	2 (0.2)
Unknown	0	6 (3.8)	11 (1.7)	3 (2.1)	20 (2.0)
Species *n* (%)					
Dog	42 (89.4)	157 (100.0)	654 (99.8)	140 (100.0)	993 (99.4)
Cat	5 (10.6)	0	1 (0.2)	0	6 (0.6)
Certainty of Agent *n* (%)					
Observed	8 (17.0)	63 (40.1)	257 (39.2)	68 (48.6)	396 (39.6)
Evidence	22 (46.8)	36 (22.9)	127 (19.4)	22 (15.7)	207 (20.7)
Suspected	2 (4.3)	11 (7.0)	56 (8.6)	16 (11.4)	85 (8.5)
Possible	14 (29.8)	42 (26.8)	210 (32.1)	33 (23.6)	299 (29.9)
Not Specific	0	1 (0.6)	1 (0.2)	1 (0.7)	3 (0.3)
Unknown	1 (2.1)	4 (2.6)	4 (0.6)	0	9 (0.9)

^a^ Designation of geographic division is based on the US Census Bureau definitions [27]. ^b^ Total percentages by column may not add up to 100 due to rounding. ^c^ More than one exposure route per call is possible.

## Data Availability

Data were procured from the ASPCA Animal Poison Control Center proprietary database and can be requested by reaching out to clientservices@aspca.org.
